# Patients’ perceived walking abilities, daily-life gait behavior and gait quality before and 3 months after total knee arthroplasty

**DOI:** 10.1007/s00402-021-03915-y

**Published:** 2021-05-06

**Authors:** Bas L. Fransen, Mirjam Pijnappels, Ise K. Butter, B. J. Burger, J. H. van Dieën, M. J. M. Hoozemans

**Affiliations:** 1 Department of Orthopaedic Surgery, CORAL-Centre for Orthopaedic Research Alkmaar, Noordwest Ziekenhuisgroep Alkmaar, Wilhelminalaan 12, 1815 JD Alkmaar, The Netherlands; 2 Department of Human Movement Sciences, Faculty of Behavioural and Movement Sciences, Vrije Universiteit Amsterdam, Amsterdam Movement Sciences, Van der Boechorststraat 9, 1081 BT Amsterdam, The Netherlands

**Keywords:** Arthroplasty, Behavior, Functional outcome, Gait analysis, Knee

## Abstract

**Introduction:**

Functional outcome and patients’ daily-life activities after total knee arthroplasty are becoming more important with a younger and more active patient population. In addition to patient-reported outcome measures (PROMs), trunk-based accelerometry has shown to be a promising method for evaluating gait function after total knee arthroplasty. The aim of this study was to evaluate daily-life perceived walking abilities, gait behavior and gait quality before and 3 months after total knee arthroplasty, using PROMs and trunk-based accelerometry.

**Materials and methods:**

A cohort of 38 patients completed questionnaires including the Oxford Knee Score and modified Gait Efficacy Scale before and 3 months after primary unilateral total knee arthroplasty. At both time points, they wore a tri-axial accelerometer at the lower back for seven consecutive days and nights. Gait behavior was calculated using gait quantity and walking speed, and multiple gait quality parameters were calculated.

**Results:**

Significant improvements were seen after 3 months in the Oxford Knee Score [median (interquartile range) 29 (10) vs 39 (8), *p* < 0.001] and modified Gait Efficacy Scale [median (interquartile range) 67 (24) vs 79 (25), *p* = 0.001]. No significant changes were observed in gait behavior (quantity and speed) or gait quality variables.

**Conclusions:**

In contrast to the significant improvements in patients’ perception of their walking abilities and PROMs, patients did not show improvements in gait behavior and gait quality. This implies that after 3 months patients’ perceived functional abilities after total knee arthroplasty do not necessarily represent their actual daily-life quantity and quality of gait, and that more focus is needed on postoperative rehabilitation to improve gait and functional behavior.

**Supplementary Information:**

The online version contains supplementary material available at 10.1007/s00402-021-03915-y.

## Introduction

With increasing numbers of total knee arthroplasty (TKA) procedures performed, a more active older population, and patients undergoing TKA surgery at a younger age, functional outcome after TKA has become increasingly relevant [1]. Particularly, gait function in terms of gait quality (e.g. gait speed, regularity and stability) and amount of ambulation appear to be important indicators for changes in physical functioning in patients with knee osteoarthritis (OA) and patients who underwent TKA surgery [2]. These changes in gait quality and amount of walking are important, since a lower quality of gait has been associated with falling [3], and a decreased gait speed with depression [4], disability [5], and even mortality [6].

Patient-Reported Outcome Measures (PROMs), which are commonly used to quantify surgical outcome, do not appear to be highly correlated with objective measures of (gait) function [7, 8]. Moreover, objective functional measures are usually assessed in a clinical setting, and earlier studies have found discrepancies between gait measurements in an optimal clinical setting and what patients actually do in daily life at home [9–12]. This implies that clinical gait function measurements may not be representative of daily life gait behavior. It follows that patient’s own perception of their walking abilities may differ from how well and how much they actually walk, especially in older adults who still constitute the largest group of patients with a TKA [13]. Therefore, to fully capture functional outcome, there is a need for additional methods to measure daily-life gait quality and quantity after TKA, allowing differentiation between patients’ perception of their gait function and their actual behavior in daily life.

The use of accelerometers, a type of inertial measurement unit (IMU), is currently emerging as an assistive tool to objectively quantify ambulation of TKA patients. It allows for both instrumented testing of functional performance in a controlled setting, and for monitoring and quantification of the amount and quality of daily life activities [14, 15]. Accelerometry measurements can be obtained in clinical and optimal settings as well as in a domestic setting, where the former can provide an objective indication of functional abilities, whereas the latter gives an impression of functional daily life behavior [9]. In an earlier study with a different patient population, we found significant improvements in several gait quality parameters (measuring amongst others stride regularity and symmetry) using trunk-based accelerometry in small bouts of gait in a controlled hospital setting [16]. Larger numbers of steps and longer bouts of walking increase the reliability of the instrumented gait measures [17, 18]. Ideally, therefore, TKA patients should be monitored for a longer period of time in a domestic setting to obtain objective and reliable measures of the quantity and quality of their daily life gait.

One-week measurements with trunk-based accelerometry have shown to be a reliable method of quantifying gait quality and behavior in other populations such as healthy older adults [19] and stroke survivors [20]. In the present prospective observational cohort study, we, therefore, used questionnaires and 1-week measurements of trunk-based accelerometry to assess knee OA patients’ perception of their walking abilities, daily life gait behavior (gait quantity and speed) and their daily life gait quality (stride regularity and symmetry) change from before to after TKA. We hypothesized that both PROMs scores and gait parameters would improve after TKA.

## Materials and methods

### Study design

A prospective observational cohort study was performed in a large teaching hospital in the Netherlands between 1 October 2017 and 30 November 2018. Patients were approached via telephone and asked to participate in the study. They then received an informed consent form through the mail (which they completed before starting measurements) together with an accelerometer and questionnaires. They were asked to wear the accelerometer on their lower back for 1 week, day and night, except during aquatic activities such as showering. Patients wore the accelerometer and completed questionnaires both before surgery and 3 months after surgery. This follow-up moment was chosen since at 3 months almost all patients are expected to have recovered to a level close to their final functional level [21]. Similarly, in total hip arthroplasty (THA) patients, most gain in gait function as measured with accelerometers in a clinical setting was found in the first 3 months after surgery [22]. Institutional Review Board approval was received from the Medical Ethical Committee Noord-Holland (number M017.011).

### Study population

Patients with grade 3–4 knee osteoarthritis, debilitating complaints, and who had previously been evaluated in the outpatient clinic and were on the waiting list for a primary TKA were eligible for inclusion. To increase external validity and to provide results indicative of the everyday function of the general orthopaedic population, we decided to include all patients who were ambulatory and willing to cooperate with study requirements regardless of comorbidity, previous surgeries or osteoarthritis in other joints besides the affected knee. Using G*Power, (Heinrich-Heine-University, Dusseldorf, Germany) [23] a population of 30–50 patients was deemed sufficient to determine a medium-sized effect of 0.5 on instrumented gait quality measures, with a correlation between repeated measures of *r* = 0.6, based on a power of 0.8 and a *p*-value of 0.05 [24]. All patients’ general characteristics such as age, gender, body heights, body mass index (BMI), smoking status, and American Society of Anesthesiologists (ASA) classification were collected from their patient files.

### TKA procedure

All patients received a Genesis II fixed-bearing total knee prosthesis (Smith & Nephew, London, United Kingdom), with a fast-track protocol with local infiltration anesthesia, early mobilization and short-acting opiates only when necessary. Procedures were performed by a team of eight surgeons, each of whom performs at least several dozens of TKA procedures per year. Patients were discharged home when they were able to ambulate independently with crutches or other walking aids or were discharged to a rehabilitation center. They were prescribed a standard physical therapy regimen, which they performed with their own physical therapist.

### Assessment of gait function

To evaluate patients’ gait function before and after TKA, three aspects of gait were analyzed: patients’ perception about their own walking abilities, gait behavior (quantity and speed), and gait quality (regularity and stability).

For quantification of patients’ perception of gait, we used The Dutch version of the modified Gait Efficacy Scale (mGES) [25]. It comprises 10 questions about daily walking tasks, with patients indicating how confident they feel regarding executing each task on a scale from 1 to 10 points. The total score ranges from 0 to 100, with 100 indicating full confidence in all tasks. Additionally, patients were asked to complete a questionnaire before surgery and 3 months postoperatively. The main PROM was the Oxford Knee Score (OKS) [26], a 12-item questionnaire with scores ranging from 0 (worst) to 48 (best). The questionnaire also included a numeric rating scale (NRS) for pain, the short version of the Knee Osteoarthritis Outcome Scale (KOOS-PS) [27], the EuroQol 5D (EQ-5D) [28], and the High Activity Arthroplasty Score (HAAS) [29].

**Fig. 1:**
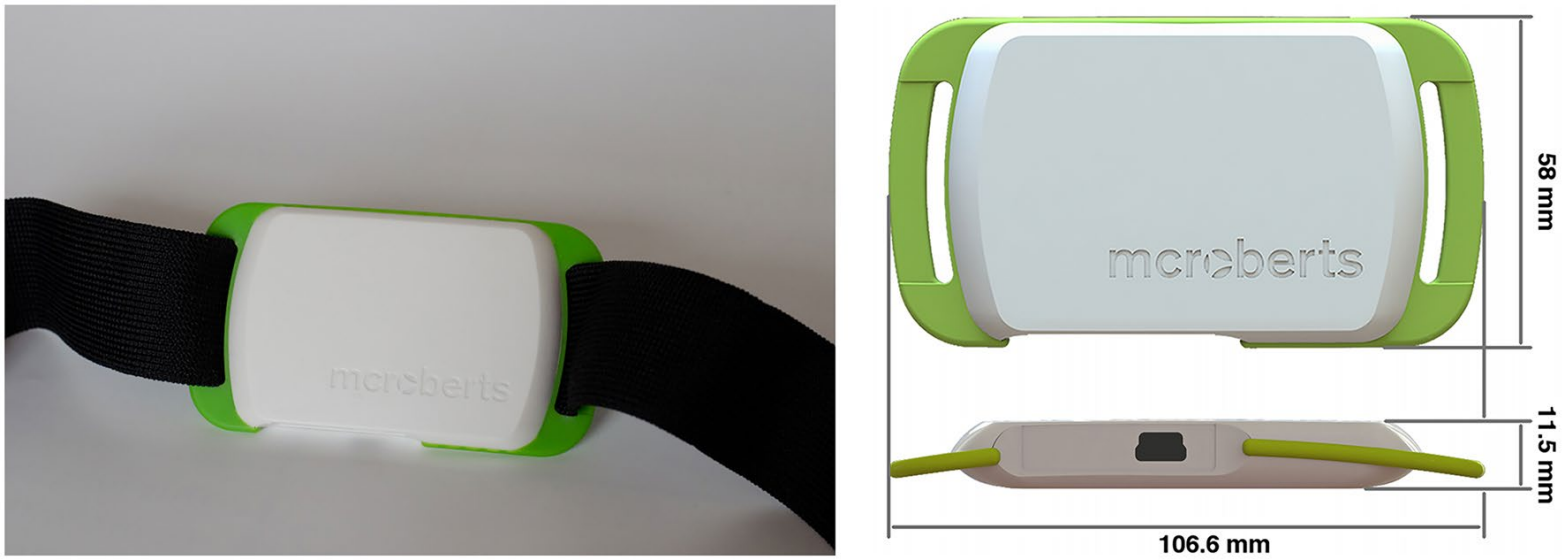
This Movemonitor accelerometer was worn by patients 1 week before and after total knee arthroplasty

Daily life physical activity was assessed during seven consecutive days using a tri-axial trunk-worn accelerometer (Fig. 1) (MoveMonitor, McRoberts, The Hague, The Netherlands). All patients received standardized written instructions on how to wear the device. Accelerations were measured in three directions: anterior–posterior (AP), medial–lateral (ML), and vertical (VT) using a range from − 6 to 6 g, with the sample rate set to 100 samples/s. Patients were instructed to wear the device at their lower back at the level of the fifth lumbar vertebrae using a Velcro belt. Accelerometer data were analyzed using MATLAB (Mathworks, Natick, USA). We used previously validated algorithms [20] to determine epochs of 8 s or more in which continuous gait was detected.

To express gait behavior, first the average number of 8-s epochs per day that the accelerometer was worn was calculated as the amount of gait. If patients reported they had inadvertently been unable to wear the accelerometer for the whole week, the actual number of days the device was worn was noted and the number of epochs per day corrected. Second, gait speed was calculated from the accelerometer data by using the leg length of patients (calculated as 53% from their body height [30]). In a previous study, we established three factors representing three different, prominent aspects of gait quality in TKA patients: AP/VT gait quality, ML gait quality, and Symmetry [16]. Therefore, in the current study we calculated the most representative variable of each of these three factors (stride regularity-VT for AP/VT gait quality, stride regularity-ML for ML gait quality, and harmonic ratio-AP for symmetry) for each of the 8 s epochs of daily-life gait. Because of the longer bouts of ambulation in the current study, additional gait quality parameters that describe the regularity and stability of the gait pattern could be calculated that were not used in the previous study: sample entropy (SE) [31] and the local divergence exponents (LDE) [32] calculated using the methods described by Wolf [33] and Rosenstein [34].

### Statistical analysis

Statistical analysis was done using IBM SPSS statistics, version 23 (IBM, Armonk, USA). For all questionnaire, gait behavior, and gait quality variables, the normality of the differences between the two assessments (before and after TKA) was checked by visual inspection of their *q*–*q* plot and box plot. A Shapiro–Wilks test was carried out on the differences. If the differences were from a normally distributed population, a paired *t*-test was used to determine if there was a significant difference between the two assessments. If the differences were not from a normal distribution, a Wilcoxon signed-rank test was used to determine if there was a significant difference between the two assessments. The gait quality parameters that were not included in the factor analysis of the previous study, i.e. SE and LDE, were also tested separately.

## Results

### Study population

**Fig. 2:**
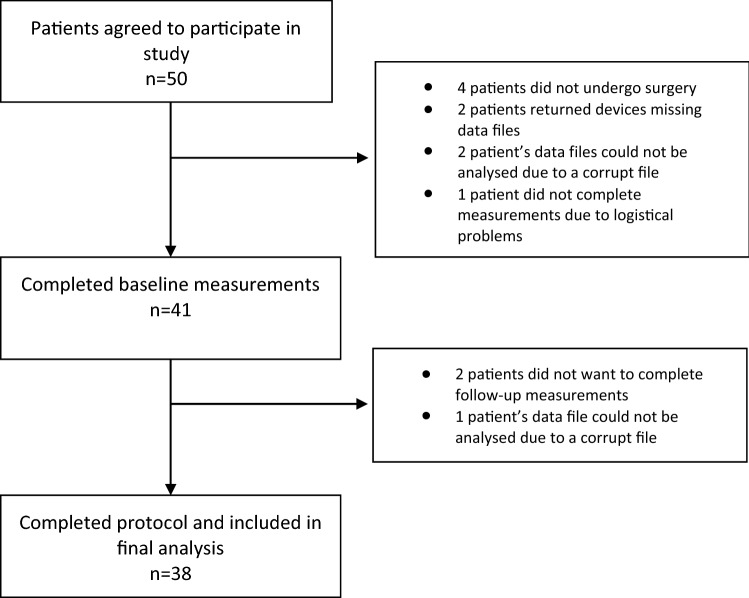
Flow chart of the study population

After being approached by phone, 50 patients agreed to participate in the study and completed the informed consent form. Out of these, a total of 38 patients completed the entire protocol and were included in the analysis. All patient characteristics are reported in Table 1, and a flow chart of the inclusion of the study population is shown in Fig. 2. Of the 12 patients that did not complete the protocol, five dropped out due to missing gait assessment because of a device malfunction, four patients had their surgery postponed or cancelled, two patients did not want to partake in follow-up measurements, and one patient did not receive the accelerometer due to logistical problems.

**Table 1:** Patient characteristics

Patient characteristics				
	Mean	Min	Max	SD
Age (years)	69	58	83	7
BMI (kg/m^2^)	29	22	36	4
	Number			
Gender				
Female	18 (47%)			
Male	20 (53%)			
ASA classification				
1	4 (11%)			
2	28 (74%)			
3	6 (15%)			
4	0			
Smoking status				
Yes	2 (5%)			
No	25 (66%)			
Quit	10 (26%)			
No answer given	1 (3%)			
Alignment				
Varus	31 (81%)			
Valgus	6 (16%)			
Neutral	1 (3%)			
TKA side				
Left	14 (37%)			
Right	24 (63%)

### Gait function

Patients’ perceived ability of gait function was measured with the mGES and PROMs questionnaires. The mGES showed a significant increase after 3 months [median (IQR) 67 (24) vs 79 (25), *p* = 0.001], indicating that patients felt more confident after TKA. The postoperative score on OKS showed a statistically significant increase [med (IQR) 29 (10) vs 38.5 (8), *p* < 0.001], which indicated better outcome after surgery as reported by patients themselves. When looking at gait behavior, the quantity of gait as measured with the median number of epochs per day was four epochs higher after 3 months, which was not statistically significant [med (IQR) 219 (179) vs 223 (123), *p* = 0.421]. In addition, there was no significant difference in gait speed between before surgery and 3 months after surgery [med (IQR) 0.85 (0.03) vs 0.79 (0.01) m/s, *p* = 0.126]. Of the three key gait quality variables and the dynamic regularity and stability measures (SE and LDE) tested, none showed statistically significant differences between the pre- and postoperative measurements (Table 2). Values before and after TKA of all questionnaire, gait behavior, and daily life gait quality measures are presented in Supplementary Table 1.

**Table 2:** Results of statistical tests of key variables

	Baseline	3 months follow-up	*p*-value
Perceived ability			
mGES (0–100)	67 (24)	79 (25)	0.001
PROMs			
OKS (0–48)	29 (9.5)	38.5 (8.25)	< 0.001
Gait behavior			
Quantity (number of 8 s epochs per day)	219 (179)	223 (123)	0.421
Gait speed (m/s)	0.85 (0.03)	0.79 (0.1)	0.126
Gait quality			
AP/VT gait quality (stride regularity-VT)	0.52 (0.1)	0.50 (0.1)	0.868
ML gait quality (stride regularity-ML)	0.42 (0.1)	0.44 (0.1)	0.073
Symmetry (harmonic ratio-AP)	1.27 (0.2)	1.25 (0.2)	0.607
Sample entropy VT	0.23 (0.1)	0.23 (0.1)	0.091
Sample entropy ML	0.30 (0.1)	0.31 (0.1)	0.936
Sample entropy AP	0.26 (0.1)	0.26 (0.1)	0.856
Local divergence exponent wolf VT (s-1)	1.60 (0.3)	1.65 (0.2)	0.658
Local divergence exponent wolf ML (s-1)	1.74 (0.2)	1.70 (0.2)	0.086
Local divergence exponent wolf AP (s-1)	1.70 (0.2)	1.64 (0.2)	0.086
Local divergence exponent rosenstein VT (s-1)	0.78 (0.1)	0.79 (0.2)	0.226
Local divergence exponent rosenstein ML (s-1)	0.66 (0.1)	0.66 (0.1)	0.845
Local divergence exponent rosenstein AP (s-1)	0.67 (0.1)	0.65 (0.1)	0.081

Data in Median (IQR), *VT* vertical, *ML* medial–lateral, *AP* anterior–posterior, *mGES* modified Gait Efficacy Scale, *PROMs* patient reported outcome measures, *OKS* Oxford Knee Score

## Discussion

We followed a cohort of 38 patients undergoing primary TKA prospectively with the aim of examining patients’ perceived walking abilities, gait behavior, and gait quality in a daily-life, domestic setting up to 3 months after surgery, using trunk-based accelerometry. Data were collected on perceived abilities using several PROMs and daily-life gait quantity and quality using instrumented gait measurements with accelerometers. We found that even though patients’ perception about their walking abilities and their OKS scores generally improved, this was not reflected in objective measures of the quantity and quality of daily-life gait function.

### PROMs and perceived walking abilities

Both the OKS and mGES showed significant increases 3 months after surgery, indicating that patients subjectively reported that their surgery was beneficial improved their gait efficacy. No minimally clinically important difference (MCID) or patient acceptable symptomatic state (PASS) have been published for the mGES, making it challenging to determine the clinical relevance of these findings. The psychometric properties, however, have been well established [35, 36]. The improvement found in OKS scores in the current study well exceeds the known MCID [37].

Earlier research suggested that an increased score on the mGES can be predictive of physical activity after TKA [38]. Therefore, the fact that in the current study patients did neither increase the amount nor speed of walking, nor improve their gait quality, is surprising. This implies that patients did not alter their gait behavior after surgery, even with more confidence in walking. It is feasible that once back home after their hospital stay, patients continued their old behavioral patterns of walking. This could be a target for postoperative rehabilitation, and provides an argument for more attention to the behavioral/perceptual aspects of patients’ rehabilitation after TKA, to supplement the functional training aimed at a range of motion and muscle strength.

### Gait behavior

In contrast to our expectations, the average number of walking epochs per day did not significantly increase in our patients. Similarly, other studies also did not find any changes in physical activity levels 1 month after TKA in the number of steps [15], after 6 months in the average amount of activity [39], and even at almost 2 years after TKA surgery in the average amount or intensity of activity [40]. When compared to healthy age-matched control patients, TKA patients appear to remain below or at most at similar levels of activity [41]. There are indications that the quantity of walking is correlated with patient satisfaction after TKA [42]. The fact that gait quantity does not improve in the first 3 months after TKA surgery could therefore be considered a possible target for increasing satisfaction rates in TKA patients. However, as patient satisfaction is strongly associated with other factors [43], the effect of increasing gait quantity on improving patient satisfaction is probably limited.

Gait speed did not change significantly after surgery in the present study. The finding that patients do not walk faster after TKA is surprising and very relevant since a higher gait speed has been associated with better gait quality and a lower risk of falling [44]. Furthermore, lower gait speed has even been shown to be a predictive factor in patients’ hazard ratio on mortality [6]. Since targeted therapy can help patients achieve higher gait speeds [45], postoperative rehabilitation after TKA could help to address the lack of increase in daily life gait speed found in this study.

### Gait quality

Our previous study using trunk-based accelerometry showed significant improvements in gait quality parameters 1 year after TKA in a controlled setting [16]. In contrast to what would be expected based on these previous results as well as TKA outcome measured with PROMs [21], 3 months after surgery the gait quality variables tested in the current study did not show statistically significant differences compared to baseline. One explanation for these findings could be that gait quality measured with accelerometers differs between a controlled clinical setting and daily life gait [9]. This raises the question whether studies that evaluate gait quality only in a clinical setting assess improvement of function and performance in optimal circumstances, and/or whether patients are motivated to perform better after surgery when observed by surgeons or researchers. For future studies, it would be interesting to have gait quality measurements of the same patients both in a clinical setting and in a daily life domestic setting at 3 months and 1 year after surgery. If there is indeed a discrepancy between the clinical and domestic setting, demonstrating this to patients could provide treating physicians and therapists with a way to motivate patients to walk more and better in their home environment. Another reason for the lack of significant differences in gait quality variables could be that patients have altered their walking behavior during their time suffering from knee OA, and that 3 months is not enough for patients to significantly readjust their behavior and thereby their gait quality (and quantity) in daily-life. In contrast to positive effects on gait function 3 months after THA [22], there are other studies that also found insufficient or no improvement of gait quality 3 months after TKA. For example, Alice and coworkers found that knee function and gait velocity had not improved to satisfactory levels 3 months after TKA as measured with 3D motion analysis in a clinical setting [46].

### Strengths and limitations

A strength of our study was the high reliability of the daily life gait characteristics, as patients wore the accelerometers at home, day and night, for 7 days. The consensus is that increasing the number of bouts of gait and, thereby, the number of steps measured, increases the reliability of gait parameters [17, 18]. Second, external validity was increased through including a heterogeneous sample of patients that had an indication for primary unilateral TKA.

We added several gait quality parameters that were not part of the factor analysis of our previous study, possibly increasing the chance of a type I error. However, since these parameters did also not show a statistically significant difference, this does not seem to be an issue.

Several patients (*n* = 12: 24%) did not complete the protocol, because of device malfunctions, logistical difficulties, postponed surgeries and/or refusal of follow-up measurements. We could not find a pattern in which patients declined to finish the protocol in a dropout analysis, and it does therefore not appear to influence the generalizability of our results. Nonetheless, the fact that 24% of enrolled patients did not complete the protocol should be taken into account when interpreting the results of this study. A comparison of baseline characteristics between patients who completed the protocol and those who did not is can be found in Supplementary Table 2.

Of three patients, it was not possible to analyze the accelerometry data. It appears that patients used the device inadequately, despite explicit instructions. For future studies, this might be prevented through the use of a smartphone app, comparable to what is already used in improving efficacy and compliance of training programs after TKA [47]. However, algorithms are at present not sufficiently accurate to validly and reliably calculate gait quality measures and gait speed from smartphone data, mostly because of the large variation in the way smartphones are carried on the body.

Information on contralateral knee joint or hip joint pain was not collected, which is a limitation of this study. Kahn et al. reported that in their analysis of physical activity before and after TKA measured with an accelerometer, comorbidities did not appear to influence the measurements in their stratified analysis [40]. They did report that this might be underpowered, so it is not possible to fully exclude the possibility that degenerative joints and previous arthroplasties in the ipsi- and contralateral leg could have influenced the walking capabilities of patients and therefore the results of this study.

## Conclusion

Our study showed that despite significant improvements in patients’ perception of their walking abilities and their PROMs scores 3 months after primary unilateral TKA, their daily-life gait behavior in terms of gait quantity, speed, and gait quality did not change. The results of this study suggest that caretakers should be aware that patients’ clinically assessed functional and perceived abilities after TKA are not necessarily representative of their actual daily activities, and vice versa. This indicates that if orthopaedic surgeons want to know how well patients function at home after TKA, objective measurements are needed to add to the data gathered using PROMs. Additionally, these results imply that improvements in quality, quantity and speed of walking after TKA could be achievable by increasing the focus of postoperative rehabilitation after TKA on improving gait and gait behavior in their domestic environment.

## Supplementary Information

Below is the link to the electronic supplementary material.

Supplementary file1 (DOCX 32 kb)

Supplementary file2 (DOCX 36 kb)

Supplementary file3 (DOCX 636 kb)

Supplementary file4 (PDF 262 kb)

Supplementary file5 (DOCX 15 kb)

Supplementary file6 (DOCX 21 kb)
