# Supplementary material for: Patients’ perceived walking abilities, daily-life gait behavior and gait quality before and 3 months after total knee arthroplasty
Source: Arch Orthop Trauma Surg. 2021 May 6;142(6):1189–96. doi: 10.1007/s00402-021-03915-y (PMC9110478; doi:10.1007/s00402-021-03915-y)
Supplement: Supplementary file 6 — Supplementary file6 (DOCX 21 kb) [file 402_2021_3915_MOESM6_ESM.docx]

Supplementary Table 1: Questionnaire, gait behavior, and daily life gait quality measures

|  | **Baseline** | **3 months follow-up** | | **p-value** |
| --- | --- | --- | --- | --- |
| Perceived ability |  |  | |  |
| mGES (0-100) | 67 (24) | 79 (25) | | .001 |
| PROMs |  |  | |  |
| OKS (0-48) | 29 (9.5) | 38.5 (8.3) | | <.001 |
| HAAS (0-18) | 6 (4) | 7 (5) | | .009 |
| VAS pain rest (0-10) | 4.5 (4) | 1 (3) | | <.001 |
| VAS pain movement (0-10) | 7 (2) | 3 (3) | | <.001 |
| KOOS-PS (100-0) | 43 (12.2) | 29.7 (13.7) | | <.001 |
| VAS QoL (0-100) | 70 (15) | 78 (19) | | .012 |
| EQ5D (0-1) | 0.78 (0.1) | 0.84 (0.2) | | .001 |
| Gait behavior |  |  | |  |
| Quantity (Number of 8 second epochs per day) | 219 (179) | 223 (123) | | .421 |
| Gait speed (meters/second) | 0.85 (0.03) | 0.79 (0.1) | | .126 |
| Gait quality |  |  | |  |
| Stride time (seconds) | 1.24 (0.1) | 1.26 (0.1) | | .001 |
| Stride time variability (seconds) | 0.06 (0.03) | 0.06 (0.03) | | .350 |
| Stride Regularity VT | 0.52 (0.1) | 0.50 (0.1) | | .868 |
| Stride Regularity ML | 0.42 (0.1) | 0.44 (0.1) | | .073 |
| Stride Regularity AP | 0.44 (0.1) | 0.47 (0.1) | | .037 |
| Sample Entropy VT | 0.23 (0.1) | 0.23 (0.1) | | .091 |
| Sample Entropy ML | 0.30 (0.1) | 0.31 (0.1) | | .936 |
| Sample Entropy AP | 0.26 (0.1) | 0.26 (0.1) | | .856 |
| Gait Symmetry (Harmonic Ratio) VT | 1.45 (0.3) | 1.40 (0.3) | | .137 |
| Gait Symmetry (Harmonic Ratio) ML | 1.34 (0.2) | 1.34 (0.2) | | .446 |
| Gait Symmetry (Harmonic Ratio) AP | 1.27 (0.2) | 1.25 (0.2) | | .607 |
| Local Divergence Exponent Wolf VT (s-1) | 1.60 (0.3) | 1.65 (0.2) | | .658 |
| Local Divergence Exponent Wolf ML (s-1) | 1.74 (0.2) | 1.70 (0.3) | | .086 |
| Local Divergence Exponent Wolf AP (s-1) | 1.70 (0.2) | 1.64 (0.2) | | .086 |
| Local Divergence Exponent Rosenstein VT (s-1) | 0.78 (0.1) | 0.79 (0.2) | | .226 |
| Local Divergence Exponent Rosenstein ML (s-1) | 0.66 (0.1) | 0.66 (0.1) | | .845 |
| Local Divergence Exponent Rosenstein AP (s-1) | 0.67 (0.1) | 0.65 (0.1) | | .081 |
| Low Frequency percentage VT < 0.7 Hz | 0.22 (0.1) | 0.24 (0.1) | | .455 |
| Low Frequency percentage ML < 10 Hz | 89.8 (7.6) | 91.1 (5.6) | | .032 |
| Low Frequency percentage AP < 0.7 Hz | 3.37 (2.8) | 3.60 (2.1) | | .500 |
| Gait Smoothness (Index of Harmonicity) VT | 0.47 (0.2) | 0.40 (0.2) | | .020 |
| Gait Smoothness (Index of Harmonicity) ML | 0.30 (0.1) | 0.31 (0.2) | | .149 |
| Gait Smoothness (Index of Harmonicity) AP | 0.56 (0.1) | 0.61 (0.2) | | .036 |
| Dominant Frequency's Amplitude VT | 0.45 (0.1) | 0.39 (0.2) | | .005 |
| Dominant Frequency's Amplitude ML | 0.31 (0.1) | 0.34 (0.1) | | .060 |
| Dominant Frequency's Amplitude AP | 0.43 (0.1) | 0.46 (0.1) | | .035 |
| *Data in Median (IQR); VT=vertical; ML=medial-lateral; AP=anterior-posterior; GES=Gait Efficacy Scale; HAAS=High Activity Arthroplasty Score; VAS=Visual Analogue Scale;* *KOOS=Knee Osteoarthritis Outcome Scale; QoL=Quality of Life; OKS=Oxford Knee Score* | | |  |  |

Supplementary table 2: Patient characteristics included and excluded patients

| **Patient characteristics** | **Included (N=38)** | **Excluded (N=12)** | **p-value** |
| --- | --- | --- | --- |
|  | **Mean (SD)** | **Mean (SD)** |  |
| Age (years) | 69 (7) | 70 (7) | 0.706 |
| BMI (kilogram/meter^2^) | 29 (4) | 29 (5) | 0.905 |
|  |  |  |  |
|  | **Number** | **Number** |  |
| Gender |  |  |  |
| Female | 18 (47%) | 8(67%) | 0.298 |
| Male | 20 (53%) | 4(33%) |  |
|  |  |  |  |
| ASA classification |  |  |  |
| 1 | 4 (11%) | 1 (8%) | 0.965 |
| 2 | 28 (74%) | 6 (50%) |  |
| 3 | 6 (15%) | 1 (8%) |  |
| 4 | 0 | 0 |  |
| Missing | 0 | 4 (34%) |  |
|  |  |  |  |
| Smoking status |  |  |  |
| Yes | 2 (5%) | 1 (8%) | 0.766 |
| No | 25 (66%) | 2 (17%) |  |
| Quit | 10 (26%) | 5 (42%) |  |
| Missing | 1 (3%) | 4 (33%) |  |
|  |  |  |  |
| Alignment |  |  |  |
| Varus | 31 (81%) | 7 (58%) | 0.131 |
| Valgus | 6 (16%) | 3 (25%) |  |
| Neutral | 1 (3%) | 2 (17%) |  |
|  |  |  |  |
| TKA Side |  |  |  |
| Left | 14 (37%) | 8(67%) | 0.026 |
| Right | 24 (63%) | 4(33%) |  |
